# Evaluation of the quality of lentic ecosystems in Romania by a GIS based WRASTIC model

**DOI:** 10.1038/s41598-021-84802-9

**Published:** 2021-03-08

**Authors:** Mihaita-Iulian Niculae, Sorin Avram, Ana-Maria Corpade, Silvia Dedu, Carmen Adriana Gheorghe, Ionut Silviu Pascu, Irina Ontel, Steliana Rodino

**Affiliations:** 1 Centre for Environmental Research and Impact Studies, University of Bucharest, 1st Nicolae Balcescu Blvd., 010041 Bucharest, Romania; 2 National Institute for Economic Research “Costin C. Kiritescu” (INCE), Romanian Academy, 13 September Street, No 13, Bucharest, Romania; 3 Department of Geography, University of Craiova, Al.I. Cuza Street, No 13, Craiova, Romania; 4 Faculty of Geography, Babeş-Bolyai University, Clinicilor Street, No 5-7, Cluj-Napoca, Romania; 5 Department of Applied Mathematics, Bucharest University of Economic Studies, 6 Romana Sq., District 1, Bucharest, Romania; 6 Department of Forest Monitoring, National Institute for Research and Development in Forestry “Marin Drăcea”, 128 Eroilor Blvd., 077190 Voluntari, Ilfov Romania; 7 Faculty of Silviculture and Forest Engineering, Transilvania University of Brasov, Eroilor Blvd., No 29, 500036 Brasov, Romania; 8 National Meteorological Administration, Bucuresti-Ploiesti Street, No.97, Sector 1, Bucharest, Romania; 9 National Institute of Research and Development for Biological Sciences, Spl. Independentei, nr 296, Bucharest, Romania; 10 Institute of Research for Agriculture Economy and Rural Development, Bd. Marasti, nr 61, Bucharest, Romania

**Keywords:** Ecology, Environmental sciences

## Abstract

Globally, ecosystems are constantly degrading as a result of pressures derived from human activities and climate change. For working towards the restoration of the natural balance, it is necessary to evaluate the deviations induced in the ecosystems, to identify where the changes took place, to know what is their amplitude and to decide where it is possible to get involved. Many aquatic ecosystems are depreciated and their restoration is often difficult. Development of appropriate assessment methodologies will improve the decision-making process in public policies for environmental protection and conservation of biodiversity. This study presents an assessment of the degradation level of lentic ecosystems in Romania, performed through a multi-criteria analysis. An extension of the WRASTIC index (Wastewater-Recreational-Agricultural-Size-Transportations-Indutrial-Cover) was generated, namely WRASTIC-HI. The new index was obtained by including values derived from the Potential Pollutant Load index. The analysis showed that 13% of the evaluated lakes are natural, 56.5% are semi-degraded and 30.5% are degraded. The proposed methodology allows to determine the spatial distribution of the degradation sources and to calculate the corresponding indicators. The results obtained provide a useful tool for diagnostic step that can be used as a cornerstone to further identification of environmental conflicts and proposals for improvement of the ecological status of the lentic ecosystems.

## Introduction

A lentic ecosystem is a system which includes living organisms (plants, animals, microorganisms) together with their physical environment, respectively the freshwater body^1–3^. Intensive human activities, such as agriculture, development of infrastructure and deforestation produce pressures on the ecosystems, altering their conditions. It was demonstrated that the aquatic ecosystem components are at high risk as a result of human activities^4,5^. Nevertheless, lentic ecosystems are affected by climate change, which in turn is determined by both human activities^6^ and natural factors. The effects of climate change on lentic ecosystems are manifested on the seasonal thermal zoning of the lake waters, with influences on the entire ecosystem^7^. The lakes capability to provide habitat for thousands of aquatic species and ecosystem services to society is threatened by size reduction, increased water salinity and/or highly altered thermal properties^8^. Diffuse pollution is affecting the largest share of lake water bodies for the aggregated broad types with worst ecological status, while point pollution is contributing to a lesser extent to decreased water quality^9^. Pollution of lentic ecosystems is generated by various external sources (waste from industrial activities, nutrient leaching from agriculture, deforestation, acid rain)^10^ and internal sources (vegetation, fauna, reduced depth etc.). Moreover, when dealing with nonpoint pollution, it becomes difficult to identify the source, volume, and impact of pollution and thus complex analysis is needed.

According to Millennium Ecosystem Assessment, 2005^2^, ecosystem degradation represents a decrease of the capacity of ecosystems to provide ecosystem services. Grizzetti^4^ highlights the importance of maintaining good ecological conditions of aquatic ecosystems to ensure the delivery of ecosystem services in the future^4^. Many aquatic ecosystems are deteriorated and their restoration is a long-term and costly process^11^. Therefore, it is preferable to maintain healthy aquatic habitats instead of having to deal with restoration. Healthy aquatic habitats depend on adaptive “management” strategies, allowing for natural recovery of lentic and lotic functions^10^. Lentic ecosystems are often undervalued in decisions related to their use, conservation or restoration^12^.

Humanity's desire to ensure their well-being aside and concerns about protecting the environment can lead to environmental conflicts^2,13,14^. These conflicts represent “the incompatible interaction between at least two actors aiming at the use of a natural resource, in which one of the actors is affected by the interaction, and the other ignores this harm”^15^. Integrating biodiversity protection into sectoral policy agendas, defined portfolio of actions and communicating the complex issue of biodiversity to different stakeholders, generally needs more attention^16^. Also, the general public could be more involved in the environmental activities, providing better information on environmental assessment items^17^.

The degradation of the aquatic ecosystems is associated with industrial activities, recreational activities, waste water, transportation infrastructure, irrigation and agricultural activities. Identifying the most important sources of degradation and designing the best indicators to assess the degradation state can provide a decision support tool for the managers of the lentic ecosystems. The spatial distribution of the degradation sources and the appropriate evaluation of the corresponding indicators allows to identify possible environmental conflicts which may occur, measures which need to be applied and strategies that should be implemented for improvement of the ecological status of lentic ecosystems.

This study aimed the evaluation of the degradation level of natural and semi-natural lentic ecosystems in Romania. The novelty of the work comes from the study’s general objective itself. No such study was performed up to the moment at national level, and the need to evaluate de degradation state of the lentic ecosystem was translated into a dedicated project, named “Development of administrative capacity of Ministry for Environment, Waters and Forests to implement the policy in the field of biodiversity”. The present results belong to this project as an independent activity for mapping of natural and semi-natural degraded ecosystems at national level. The innovative character of the research comes from the development of WRASTIC-HI index, which was built according to data availability. Therefore, the data presented here are representing the authors response regarding the necessity identified by stakeholders related to assessment of degradation of aquatic ecosystems in Romania.

First of all, the approach involved the delimitation and analysis of the spatial distribution of such ecosystems and the assessment of their degradation status, by using GIS techniques. The lentic ecosystems were classified in three categories as follows: degraded, semi-degraded and natural lentic ecosystems. In the second stage, relevant indicators, previously identified as determinants for assessment of environmental degradation in a multifactorial analysis^1,18,19^, were calculated. Finally, a correlation between the calculated indicators and the degradation state of lentic ecosystems was done.

## Results and discussion

A total number of 3189 lakes have been spatially delimited and analyzed. The delimitation and spatial distribution of the lakes revealed their uneven distribution within the Romanian territory. The largest share of lakes, (41.5%) are distributed within the low plains, located mainly in the South and West of the country. About 36% of the identified lakes are located in the hilly and plateau units, while 11.5% are in the mountain areas and about 11% in the Danube Delta.

**Figure 1:**
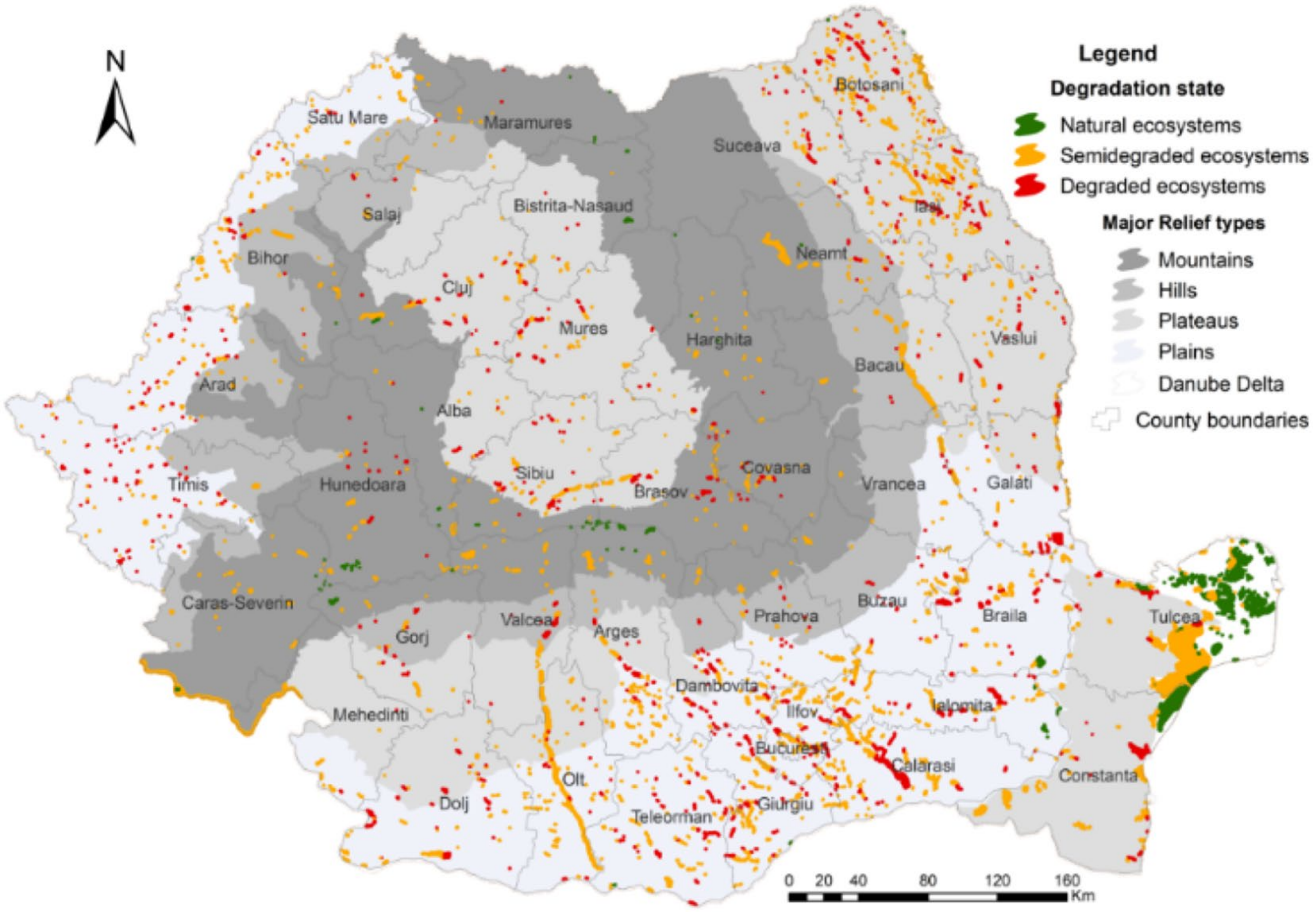
Distribution of lentic ecosystems in Romania according to the major topography units (this map was created with Arc GIS 10.5 software).

The assessment of the state of degradation of the lentic ecosystems by the GIS based extended WRASTIC model revealed that more than half (57%) of the analyzed lakes are classified as semi-degraded. These are mostly distributed in the plains (46%) and in the plateau areas (33%) (Fig. 1).

The lakes classified as degraded represent 31% of the total, mostly located in the plains (48%) and plateau (36.5%), the least part being located in the Danube Delta (about 0.5%). The lakes in the natural state represent a small share, respectively about 13%, generally located in the Danube Delta (72%) and the mountain areas (21%) (Supplementary Table 1).

**Figure 2:**
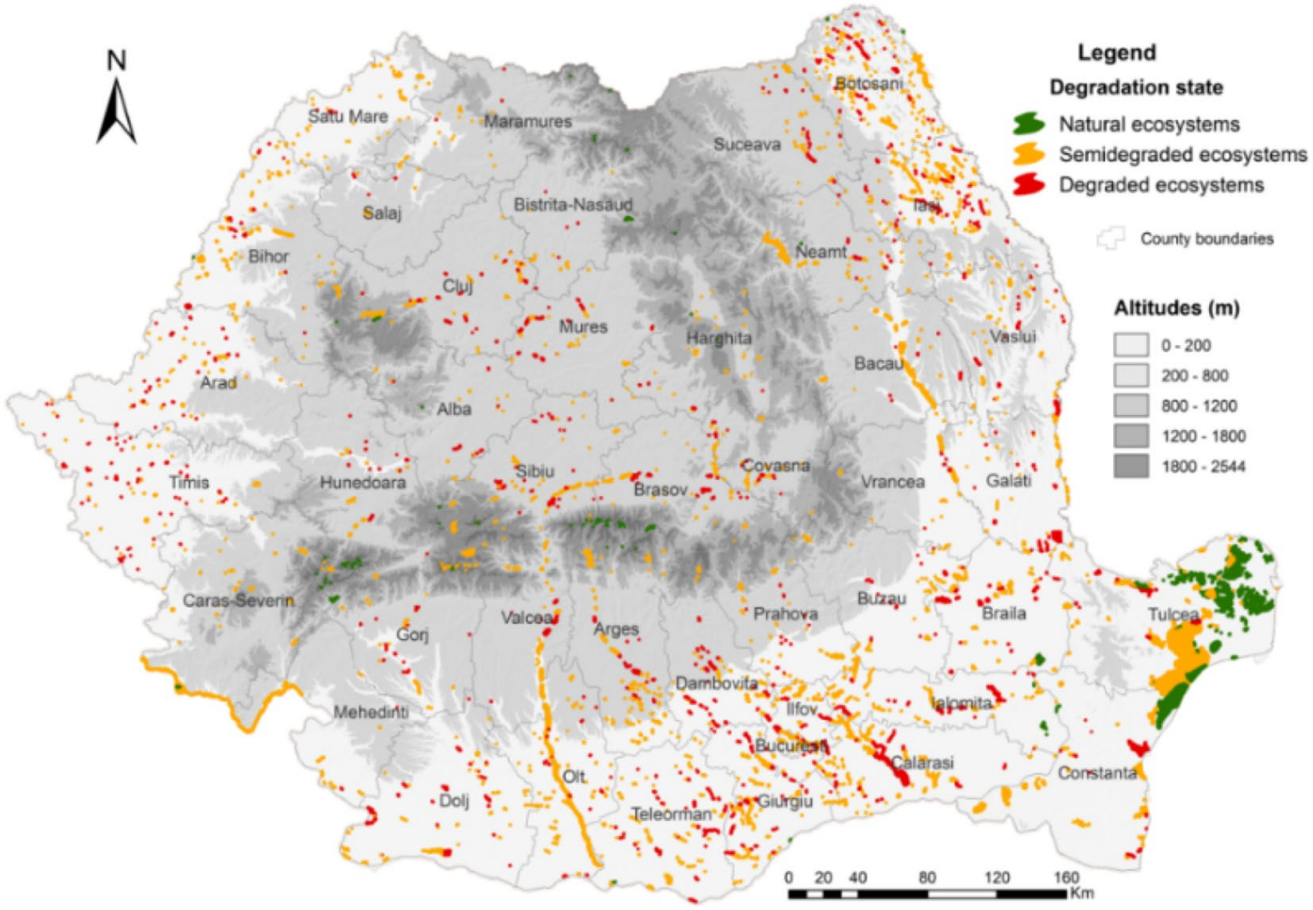
Distribution of lentic ecosystems on the Romanian territory according to the altitudinal stages (this map was created with Arc GIS 10.5 software).

Altitudinal stages represent a restrictive factor in the spatial distribution of lakes throughout the country. Most lakes, about 76%, are located at low altitudes, respectively below an altitude of 200 m. The number of lakes identified at higher altitudes, over 800 m, is reduced, representing about 8% of the total number of analyzed lakes (Fig. 2).

Regarding the natural lakes, the largest part of them is represented by natural lakes located at altitudes less than 200 m (about 83%). In the same time, about 75% of the lakes evaluated as degraded are located at altitudes less than 200 m.

**Figure 3:**
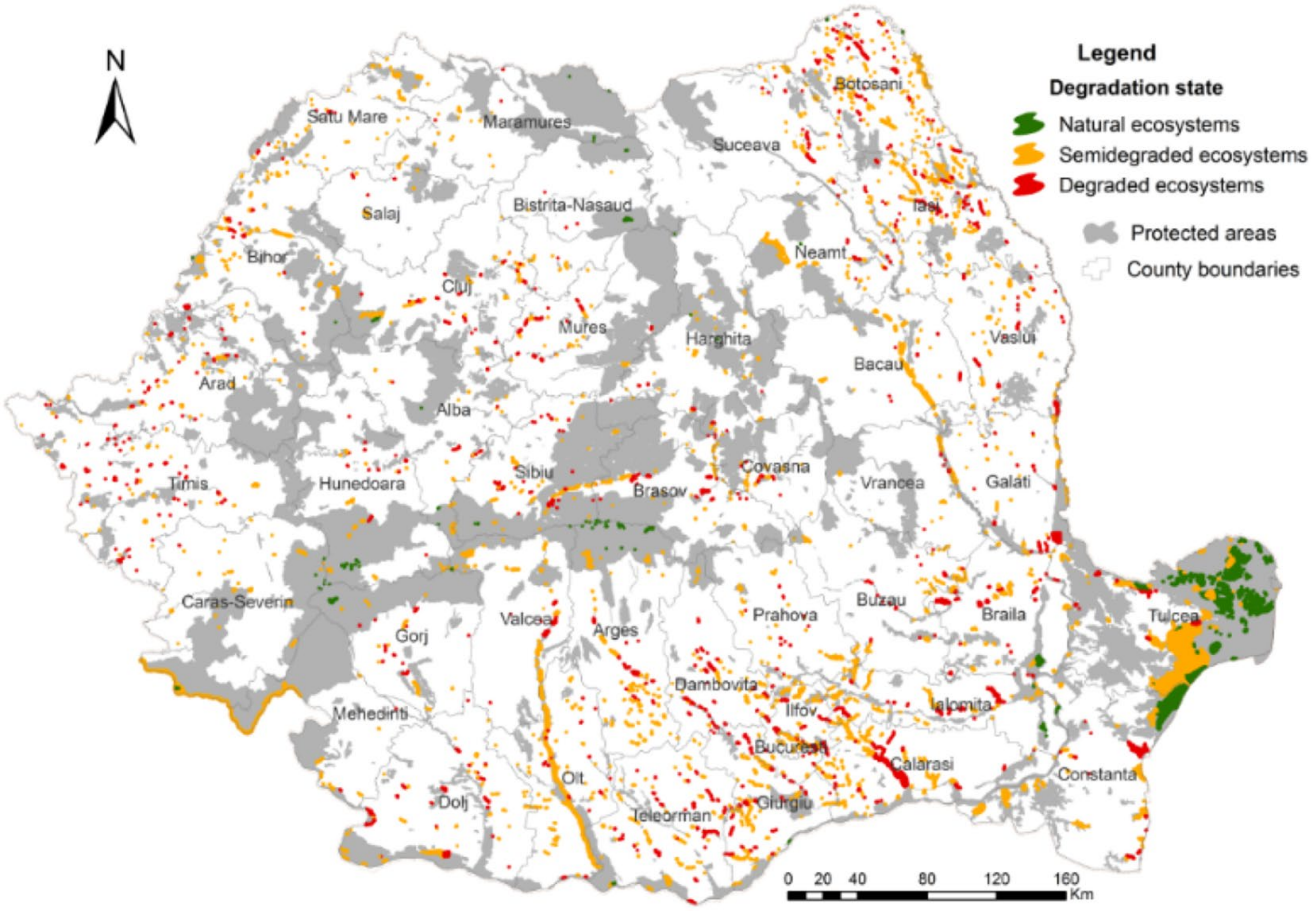
Distribution of lake categories according to the state of degradation and the relationship with protected areas of national and international interest in Romania (this map was created with Arc GIS 10.5 software).

About 98.5% of the 412 lakes in the natural state are located, partially or totally, in protected areas of national or international interest, such as national and natural parks, Ramsar sites, Biosphere reserves, UNESCO World heritage sites and Natura 2000 sites (Sites of Community Importance and Special Protection Areas) (Fig. 3.).

The percentage of semi-degraded and degraded lakes located in protected areas is lower (about 38% of the semi-degraded lakes, respectively 32.5% of the degraded) (Supplementary Table 2). The other lakes are not included in protected areas and do not own any special protection regime.

As a result of applying the methodology based on WRASTIC-HI index, only 9 lakes out of the total of 412 natural lakes are associated with industrial activities and different forms of small-scale exploitation within their hydrographic basins, while the rest of 98% do not present such activities.

The hydrographic basins of the natural lakes do not cover very large areas, 83% of them stretching on area of less than 39 square km, each. In about 97% of cases, agricultural activities in the reception basin represent below 20% of the economic activity, the irrigation percentage being low and the degree of vegetation cover being high.

Considering the degraded lakes, for a large share of them (87%) of them industrial and exploitation activities, such as mines, quarries or dumps are present within the river basin. A high percentage, about 98%, include treatment plants with different types of processing (primary or secondary) within the reception hydrographic basins. For 96% of the degraded lakes, agricultural activities and permanent irrigation activities are covering over 40% of the related basins area.

Spearman correlation showed that there is a good correlation between the degradation state and several components of the WRASTIC-HI index such as Industrial activities, Recreational activities, Wastewater, Ways of transportation, Irrigation and Agricultural activities (Table 1).

**Table 1:** Correlation between Degradation state and component indices of the WRASTIC-HI index.

			I^1^	R^1^	W^1^	S^1^	T^1^	V^1^	G^1^	A^1^
Spearman's rho	Degradation state	Correlation coefficient	.647**	.582**	.421**	− .019	.492**	.370**	.409**	.503**
		Sig. (2-taylored)	.000	.000	.000	.292	.000	.000	.000	.000
		N	3189	3189	3189	3189	3189	3189	3189	3189

^1^I = industrial activities; R = recreational activities; W = waste waters; S = size of watershed; T = ways of transportation; V = vegetation; G = irrigation; A = agricultural activities; *Correlation is significant at the 0.05 level (2-tailed), **Correlation is significant at the 0.01 level (2-tailed).

Between the state of degradation, on the one hand, and the permeability of the soil, the slope and the vegetation cover of the water lily, on the other hand, the analysis showed that there is no correlation. However, there is a weak correlation between the degradation state and Exposition (Table 2).

**Table 2:** Correlation between degradation state and the HI index variables, which belong to the WRASTIC-HI index.

			Soil permeability	Land slope	Slope aspect	Vegetation 1	Vegetation 2
Spearman's rho	Degradation state	Correlation coefficient	.036*	.024	.235**	.156**	− .090**
		Sig. (2-taylored)	.040	.167	.000	.000	.000
		N	3189	3189	3189	3189	3189

*Correlation is significant at the 0.05 level (2-tailed), **Correlation is significant at the 0.01 level (2-tailed).

The computational results indicate a negative correlation between the state of degradation, the percentage of the basin included in the protected areas and the percentage of the basin included in the protected areas of the Natura 2000 network (the probability level of 0.0001, being less than 0.05, indicates that there is a statistical significance) (Table 3).

**Table 3:** The results of the statistical analysis regarding the relationship between the state of degradation and variables within the river basin.

			Percentage of lake basin in protected areas	Number of inhabitants without access to sewage	Population density in the lake basin	Percentage of lake basin in Natura 2000 protected areas
Spearman's rho	Degradation state	Correlation coefficient	.036*	.024	.235**	.156**
		Sig. (2-taylored)	.040	.167	.000	.000
		N	3189	3189	3189	3189

*Correlation is significant at the 0.05 level (2-tailed), **Correlation is significant at the 0.01 level (2-tailed).

The statistical analysis performed shows that there is no correlation between the Degradation State and Altitude, while between the Degradation state and the Relief units there is a weak negative correlation.

The results indicate also a direct correlation between the state of degradation, the number of inhabitants without access to the sewerage and the population density in the lake basin, the correlation coefficient being 0.024, and respectively 0.235.

It is important to study of the state of the lentic ecosystems both regarding the pressures coming from human activity and the ones originating from climatic changes or other natural phenomena. However, the development of an assessment model that can be applied to all aquatic ecosystems, and lentic ecosystem in particular, is a challenge, because available data are not homogenous, each region and lake having its own particularities. Choosing the proper set of indicators that can be useful for the overall characterization of the quality of lentic ecosystems, will lead to a coherent implementation of biodiversity strategies.

To the best of our knowledge, this is the first evaluation of the quality of lentic ecosystems in Romania by multi-criteria analysis. This assessment is part of a national project for implementation of a national policy on biodiversity, as a response to EU requirements.

Over the time, the scientific literature identified various methodologies for the evaluation of the quality of water resources. Such methods include DRASTIC^20,21^, and methods derived from DRASTIC^22–24^. Other authors used digital surface model (DSM) and a point dataset as the sources of observation and target locations for Geospatial analysis of lake scenery^25^.

For integrative water quality management, Feng et al.^26^, proposed a model-based method. Their method integrated three indices derived from three models for assessment of the risk due to nutrient dynamics^26^.

A multi-attribute value theory to formulate an integrated water quality assessment method was used by Schuwirth^27^, for aggregation over multiple pollutants and time.

Another category of indexes that are used in evaluating the state of degradation of lacustrine ecosystems aim at analysis of the presence of degradation sources in supplying basin. For example, Mirzaei et al^19^ calculated WRASTIC index for assessment of pollution risk, respectively degradation sources, from the watershed that feeds a water body. Potential pollutant load index (PPL) was employed by Romanelli et al^18^ for analyzing the presence and intensity of potential pollution sources from the drainage area of several lakes, with the purpose of establishing degradation classes of the water body. In the same study, the Lake Vulnerability Index highlighted the capacity of the water body to handle the impact generated by degradation sources, taking into account parameters like slope, soil permeability or aspect of slopes^18^.

According to the characteristics of the study area, modified versions of WRASTIC index are to be found in the literature, and were implemented by using additional criteria or eliminating some parameters^28^.

Rahimi et al^29^ used WRASTIC Index for evaluation of wetland water quality. Their results revealed that the activity in adjacent wetland areas exert a large impact on wetland integrity^29^.

In another study, aquatic ecosystems pollution risk was evaluated by a combined Fuzzy-WRASTIC method. The model was validated by comparison with samples collected from the case study area. The authors concluded that the method has advantages over other methods, as it includes a wide range of drivers and parameters that influence the water quality. The results obtained pointed that areas with high contamination risk are due to the unbalanced arrangement and compact of land uses in the neighborhood of the aquatic ecosystems^30^. Using analytical survey and experimental studies Mirzaei et al^19^ investigated the pollution risk for Zayandehrud river, Iran. Agricultural, industrial activities and population centers were the main causes of pollution in the study case area^19^.

In Romania, the evaluation of aquatic ecosystems was performed by various researchers, either for a specific area or for a hydrographic basin.

For example, Rosca et al^31^ studied the impact of anthropogenic activity on water quality parameters of glacial lakes from Rodnei mountains. The factors taken in consideration were tourism and livestock. The pollution index was calculated based on three indices, targeted on heavy metal influences, namely, the heavy metal pollution index (indicating the quality of waters related to the heavy metals content), the heavy metal evaluation index (assessment of the quality of water with respect to heavy metals) and the degree of contamination (used to quantify the contamination level with the heavy metal). The physico-chemical parameters pointed a good quality of the study case lakes. The conclusion of the authors was that minor anthropic alteration and a low anthropogenic impact is exerted in these areas. The only anthropic pressure on the aquatic systems in Rodnei Mountains was reported as being exerted by grazing activities^31^.

Another paper described the assessment of actual water quality and sedimentological conditions of the Corbu lake, Western Black Sea coast. The ecological status of this lake was found to be from good to weak classes for nitrites, ammonium and phosphates, moderate for sulphates and weak for detergents^32^.

The impact of human interventions and climate changes on the hydro-chemical composition of Techirghiol lake (Romania) was recently investigated by Maftei et al^33^. The study identified a degradation of this ecosystem between 1970–1998, due to extensive irrigation in the lake region, followed by a major decrease of the lake’s salinity^33^. Physico-chemical water quality parameters of lake Brăneşti was investigated by Benciu et al^34^. The water quality parameters for the last 50 years were correlated with the anthropogenic pressure in the region. Analysis of water and soil samples in the vicinity of this lake, revealed that parameters were within legal norms for both water and soil^34^*.*

Another study presented by Dumitran et al^35^ proposed an eutrophication model for describing the ecological behavior of a eutrophic lake. The physical model was mathematically transposed to a set of equations for analysing the selected parameters linked to eutrophication state. The resulted model showed a good correlation with the measured data^35^.

It is to be noted that most of the available literature is based on the assessment of the water quality, by measurements of physico-chemical parameters, and calculation of pollution indexes. To our knowledge no extensive studies involved the study of the lentic ecosystems with respect to vulnerability and risk of pollution by using multicriterial analysis.

Generally, the precautionary approach is applied by identifying and analyzing the categories of drivers that influence the degradation of lentic ecosystems, especially in the protected areas^36–38^. Three main categories of activities that generate environmental issues have been identified within protected areas included within the Natura 2000 Sites as follows: (a) agricultural activities and forestry practice; (b) sectoral activities (industrial, commercial and tourism sectors); (c) conservation policies (management of protected areas, protection of different species, etc.)^39^. A cross-sectoral approach is needed in order to resolve medium-term environmental conflicts, thus being be able to extend the assessment towards various categories of protected areas and generating efficient policies for the management of resources^40,41^.

Identifying and analyzing the categories of conflicts that may be associated with lentic ecosystems provide the possibility of an efficient ecosystem management^22,23^.

The lakes from this case study comprise both natural lakes (glacial, karst, karst-saline, ponds, lagoons), as well as ponds accumulation lakes, with an important role in ensuring the resources of water for the population and economic activities, as well as the development and maintenance of habitats and species of community interest (birds, amphibians, reptiles, fish, etc.).

Most of the lakes resulting from the analysis as being degraded and semi-degraded are located in the plains, at low altitudes, within areas covered with agricultural lands, industrial facilities and dense transportation routes.

The lentic ecosystems characterized as being in a natural state resulting from the proposed methodology are mainly distributed in the high mountain areas and in the Danube Delta area. Thus, altitude, fragmentation of the relief and accessibility are favorable factors regarding the natural state of water bodies, including lentic ecosystems. The high number of lakes characterized as degraded or semi-degraded compared to that of natural lakes is justified by the existence of a small number of lakes located at high altitudes, over 800 m.

It is to be taken into account that the degradation state classification is directly influenced by the data used in defining WRASTIC indicators, being generally derived data, which may explain the limitation of the method from this point of view.

The lack of correlation or poor correlation resulting from the statistical analysis between the degradation state and the indicators defining the HI index (component part of the WRASTIC-HI index), respectively the permeability, the exposure and the slope, highlight the insignificant role of these parameters in determining the state of lake degradation. However, the processes of erosion and sediment transport on the surface of the basins and their accumulation in lakes can influence the water quality of the lakes, their clogging, their functionality and the services offered, which are important factors in improving the management of the analyzed lakes^42^.

The status of protected areas offers a high degree of protection by diminishing the anthropic activities and the negative effects on the lakes, being recommended that all economic activities be located outside these protected areas^43^ the basins being vulnerable to human activities. This aspect is also highlighted by the correlation between the state of degradation obtained and the percentage of the hydrographic basin existing in different categories of protected areas, between which there is a good correlation, as well as by the high number of natural lakes that are included in protected areas (~ 98%), located especially in the Danube Delta Biosphere Reserve.

The local public administrations are directly interested in the management and protection of lentic ecosystems, many lakes being included in different categories of protected areas. Increasing the number of lakes in the natural state involves identifying degraded or semi-degraded lentic ecosystems outside protected areas and carrying out ecological reconstruction activities or diminishing agricultural and industrial activities in their vicinity.

The state of the lakes may also depend on the dynamics of the hydrophilic and hydrophilic vegetation, respectively on the hedge with vegetation cover of the water lily. In our study, the statistical analysis showed that there is no correlation between the state of degradation and the Coverage with vegetation of the water lily. Thus, in this case, the state of degradation of the lentic ecosystems is not influenced by this parameter, although, in some cases, remote sensing analysis revealed the presence of excess algae and aquatic plants in both natural and semi-degraded lakes^44^.

The lakes located in the low plain areas are also affected by the eutrophication process, amplified by the reduced depth (which ensures the rapid development of algae during the summer), the contribution of nutrients due to the agricultural activities in the vicinity and the development of recreational activities.

## Conclusions

A small share (2%) of the lakes identified as being in natural state (412 lakes) resulted as being influenced by the industrial activities performed within their hydrographic basins. For approximately 97% of these lakes the agricultural sector influence was below 20%, with low irrigation activity and high vegetation cover. In the case of degraded lakes, for a percentage of 87% of them, industrial and exploitation activities were identified within their hydrographic network.

Spearman correlation showed that there is a good correlation between the degradation state and some components of the WRASTIC-HI index such as Industrial activities, Recreational activities, Wastewater, Ways of transportation, Irrigation and Agricultural activities.

The methodology used has taken into account the potential sources of degradation within the basin around the lake analyzed and is representing an alternative to the methods that involve direct laboratory analyses regarding the quality of the lake water. The relevance of the method proposed in this paper consists in identifying and analyzing the factors that generate degradation. Therefore, this method could guide the actions for reducing the negative influence of the disturbing factors.

The existence of a high number of degraded or semi-degraded lakes at national level resulted from the analysis highlights the need to implement large-scale ecological reconstruction projects, as well as the monitoring of freshwater habitats and species of community interest, according to the provisions of the European Directives assumed by Romania.

The methodology proposed in this paper can be applied on a large scale and represents an alternative to determining the quality of the lake water carried out by direct field or laboratory analysis, which involves human resources and high costs.

## Methods

The final value of the composite index, WRASTIC-HI was obtained by assigning scores and computing their corresponding weights for each criterion considered in the model. Thus, using the final values obtained, the lacustrine ecosystems have been divided into three categories, depending on the state of degradation: naturally, semi-degraded and degraded. The higher the final value of the WRASTIC-HI index, the higher is the degradation level of the analyzed lake.

In the previous work of the authors, after design and calculating the WRASTIC-HI INDEX, the methodology was validated with real data from the field. The data obtained was correlated with the existing field situation for the sample lakes, and thus resulted the classification criteria for the analyzed lakes into natural, semi-degraded and degraded lentic ecosystems. The three classes were chosen in accordance with the regeneration capacity of the ecosystem versus the need for ecosystem restoration and unlocking the resources needed for ecological reconstruction.

Natural ecosystems were classified as those where anthropic interventions are missing or insignificant and in which the values of the physical and chemical elements are unaltered or significantly altered. Semi-degraded lake ecosystems are those where anthropogenic alterations lead to a moderate disturbance of the values of physico-chemical, hydromorphological and biological indicators. Finally, the degraded ecosystems are those where anthropogenic interventions have led to a serious disturbance of the values of physico-chemical, hydromorphological and biological indicators.

### Identification and delimitation of the lentic ecosystems at national level

Copernicus Pan-European High Resolution Permanent Water Bodies database^45^ with 20 m resolution was used for the delimitation of lentic ecosystems. The data collected was compared with individually determined reference values—satellite recordings from Landsat 8 OLI/THIRS program, with a resolution of 30 m and 15 m respectively^46^. The delimitation of the permanent water bodies used as reference was performed using the Normalized Difference Water Index (NDWI)^47^ and Tasseled Cap (TCW)^48^.

A method derived from that proposed by Bangyu et al^49^ has been developed. First, the water bodies were delimited. Second, a composite index was derived using the set of specific Landsat 8 bands, using bands 5, 6 and 4 as a substitute for the red, green and blue bands. Another stage involved unsupervised classification to initially extract water bodies from the other classes, using the Iso Cluster method as part of the ArcGIS [GIS software] (Version 10.0. Redlands, CA: Environmental Systems Research Institute, Inc., 2010)^50^. Finally, the resulting data were reclassified, allowing the retention of the classes defining the water bodies and the elimination of the unfavorable classes.

### Assessment of the state of degradation of the lentic ecosystems identified at national level

GIS techniques have been used to evaluate the state of degradation of lentic ecosystems.

WRASTIC-HI index was developed by using three indicators (Supplementary Fig. 1), as follows: Potential Pollutant Load (PPL)^18^, Wastewater—Recreational—Agricultural—Size—Transportations—Industrial—Cover—Pollutant Load (WRASTIC)^51^ and Lake Vulnerability (LV)^18^. PPL and WRASTIC use information regarding the categories of land use within the basins of the lakes analyzed, identified as possible sources of degradation. LV takes into account data related to slope, exposure and soil characteristics, determining factors in the drainage of polluted water to the lentic ecosystems analyzed as accumulation points.

WRASTIC-HI was defined by the following components: Wastewater discharge (W), Recreational land use impacts (R), Agricultural land use impacts (A), Size of watershed (S), Transportation ways (T), Industrial land use impacts (I), The amount of vegetative ground Cover (C), Hazard Index (HI), which distinguishes Permeability (P), Exposition (E) and Slope (S) (Supplementary Tables 3, 4)^18,19^.

The WRASTIC-HI is computed using the following formula:

$$WRASTIC{ - }HI = \left( {Wn \times Wp + Rn \times Rp + An \times Ap + Sn \times Sp + Tn \times Tp + In \times Ip + Cn \times Cp} \right) \times \left( {Sn + En + Pn} \right)$$

where the symbols W, R, A, S, T, I, C signify the proposed criteria used for the analysis, *n* denotes the score of each criterion, *p* stands for the weight of each criterion, *S* represents the terrain slope, *E* denotes the exposure and *P* the permeability of the soil.

The score of the index for each lake was computed based on the data collected from the databases, and correlated with sample lakes used for validation of the methodology^1^.

The process of establishing the degree of degradation involves several intermediate analyzes, respectively obtaining specific data from complex datasets (Supplementary Table 5).

For the calculation of the soil permeability, the Pedological Map of Romania, scale 1: 200,000, was used.

The result is stored in the database required for the iterative calculation model of WRASTIC-HI. It will use the Clip function to extract soil-specific information from the same region as the previous clues.

Based on the calculated data required for LV, its value will be calculated as the sum of the three rasters and stored for assigning WRASTIC values.

Soil permeability was derived from the textural classes associated with the soil types represented in the 1: 200,000 pedological map (Supplementary Table 6).

The slope exposure is calculated from a hybrid numerical model that combines the SRTM and ASTER GDEM data by weight, characterized by a spatial resolution of approximately 25–25 m.

Based on the maximum rate of change of value between each cell and the neighboring cells, the slope direction is calculated.

The interval 0‒360 was reclassified, obtaining exposures corresponding to the cardinal points (Supplementary Table 7).

Depending on the orientation of the shore, the nine classes are reduced to three (5—exposure favorable to the accumulation of pollutants in the lake, 3—exposure with neutral effect on the accumulation of pollutants in the lake and 1—exposure unfavorable to the accumulation of pollutants in the lake).

To obtain the Hazard Index indicator, the values of the slope and the permeability were summed up rasterially, and then they are reclassified to show the effect on the accumulation of pollutants (Supplementary Table 8).

The result of the rasterial sum is then analyzed from the perspective of the slope exposition, obtaining the HI value (Supplementary Table 9).

The ArcGis software^50^ and the Python programming language have been used to compute the selected indicators and to develop an automatic method for assessing the impact of all factors involved in the model and generating an independent degradation index^1^. Also, the ModelBuilder programming environment has been employed. The iterative structure of the resulting operations has been grouped into ArcToolbox and the data used by this toolbox have been stored in a .gdb type database, including vector and raster data files.

The assessment of the HI Index is achieved by computing the exposure and the slope, based on data obtained from digital elevation model-DEM (European Environmental Agency)^52^. The development of the WRASTIC Index is performed by generating the network of hydrographic basins based on the numerical model. For evaluation of the vegetation cover share the CLC database—level 3 was used together with land coverage data extracted from the APIA database, available from the Romanian Agency for Payments and Intervention in Agriculture (APIA)^53,54^. LPIS database was also used for data regarding the impact of the activities from the agricultural sector. Regarding the impact of recreational activities, data from the management plans of the protected areas have been collected.

The distribution of the protected areas categories used in the study have been extracted from the Ministry of the Environment database^55^, and the limits of the major topography units correspond to the vector data related to the topographical map of Romania^56^.

Weighting, rating and ranking implied review of literature data, field data and expert opinion. The methodology applied, the score of the index and the weight computed was validated in a previous study by applying this rationale to a sample of lakes for which real field data were available. The results of index calculation corresponded in all case studies to the real field situation, highlighting thus accuracy of the assessing process and increased advantages of assessment’s automation^1^.

### Statistical analysis

To analyze the correlation between the degradation state and the resulting quantitative values for the indicators that compose the WRASTIC-HI index, the Spearman coefficient has been calculated. Spearman correlation has been used to evaluate the relationships between environmental variables and water quality, providing a range of quantitative information. Statistical data analysis has been performed using IBM SPSS Statistics 22 software^57^.

## Supplementary Information

Supplementary Information
