# Supplementary material for: Evaluation of the quality of lentic ecosystems in Romania by a GIS based WRASTIC model
Source: Sci Rep. 2021 Mar 8;11:5361. doi: 10.1038/s41598-021-84802-9 (PMC7970884; doi:10.1038/s41598-021-84802-9)
Supplement: Supplementary file 1 — Supplementary Information [file 41598_2021_84802_MOESM1_ESM.docx]

**Supplementary Materials**

**EVALUATION OF THE QUALITY OF LENTIC ECOSYSTEMS IN ROMANIA BY A GIS BASED WRASTIC MODEL**

Mihaita-Iulian Niculae^1^, Sorin Avram^2,3^, Ana-Maria Corpade^4^, Silvia Dedu^5^, Carmen Adriana Gheorghe^2^, Ionut Silviu Pascu ^6,7^, Irina Ontel^8^, Steliana Rodino^9,10*^

^1^ University of Bucharest, Centre for Environmental Research and Impact Studies;1st Nicolae Balcescu Blvd., Bucharest, Romania

^2^ National Institute for Economic Research “Costin C. Kiritescu” (INCE), Romanian Academy, 13 September Street, No 13, Bucharest, Romania

^3^ University of Craiova, Department of Geography, Al.I. Cuza Street, No 13, Craiova, Romania

^4^ Babeş-Bolyai University, Faculty of Geography, Clinicilor Street, No 5-7, Cluj-Napoca, Romania

^5^Bucharest University of Economic Studies, Department of Applied Mathematics, 6 Romana Sq., District 1, Bucharest, Romania

^6^ National Institute for Research and Development in Forestry "Marin Drăcea", Department of Forest Monitoring, Address: 128 Eroilor Blvd., Voluntari, Ilfov, Romania

^7^Transilvania University of Brasov, Faculty of Silviculture and Forest Engineering, Eroilor Blvd., No 29, Brasov, Romania

^8^National Meteorological Administration, Bucuresti-Ploiesti Street, No.97, Sector 1, Bucharest, Romania

^9^National Institute of Research and Development for Biological Sciences, Spl. Independentei, nr 296, Bucharest, Romania

^10^Institute of Research for Agriculture Economy and Rural Development, Bd. Marasti, nr 61, Bucharest, Romania

* Correspondence and requests for materials should be addressed to: steliana.rodino@yahoo.com; avram.sorin32@gmail.com

**Table 1.** Number of lakes depending on the state of degradation and the main units of relief.

|  | Total number  of lakes | Plains | Mountains | Plateaus | Hills and Subcarpathians | Danube Delta |
| --- | --- | --- | --- | --- | --- | --- |
| Total number of lakes,  *from which:* | 3189 | 1326 | 363 | 954 | 189 | 357 |
| *Natural ecosystems* | 412 | 29 | 85 | 2 | 0 | 296 |
| *Semi-degraded ecosystems* | 1805 | 830 | 207 | 596 | 115 | 57 |
| *Degraded ecosystems* | 972 | 467 | 71 | 356 | 74 | 4 |

**Table 2.** The categories of lakes located in the network of protected areas in Romania

|  | Total number of lakes | Protected areas | |
| --- | --- | --- | --- |
|  |  | **All Protected areas** | **Natura 2000 network** |
| Lentic ecosystems,  *from which* | 3189 | 1413 | 1407 |
| *Natural ecosystems* | 412 | 407 | 407 |
| *Semi-degraded ecosystems* | 1805 | 690 | 685 |
| *Degraded ecosystems* | 972 | 316 | 315 |


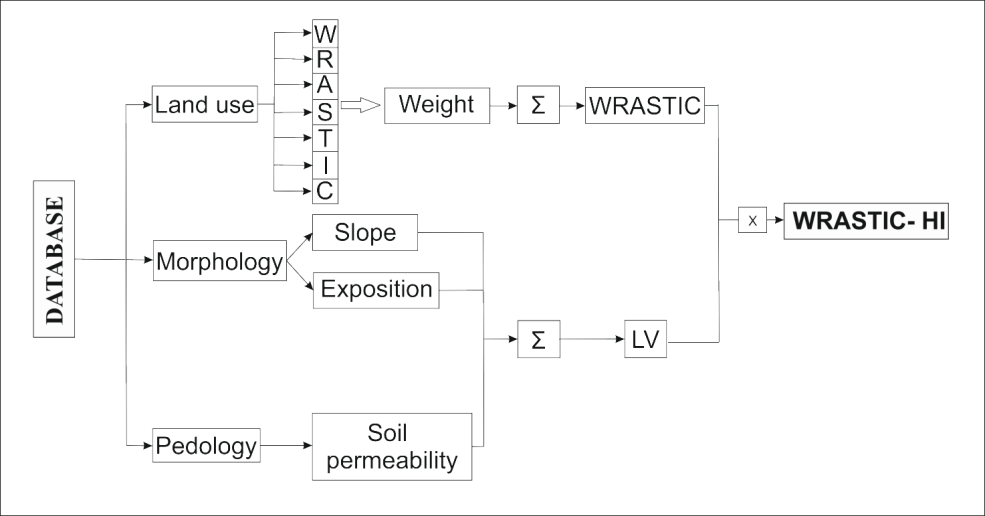


**Figure 1.** The computation model for the WRASTIC-HI index, derived from aggregating the three indicators

**Table 3**. Criteria (indicators) used for calculation of the WRASTIC index

| Category of use | Subcategory | Interval | Score | Weight |
| --- | --- | --- | --- | --- |
| Waste water (W) | Aggregating cores | Natural Breaks (ArcGIS) | 1 – 4 | 3 |
|  | Treatment plants | Primary processing | 3 |  |
|  |  | Secondary processing | 2 |  |
|  |  | Tertiary processing | 1 |  |
| Recreational activities (R) | Aquatic sports | Motorized | 5 | 3 |
|  |  | Non-motorized | 4 |  |
|  | Access | Auto | 3 |  |
|  |  | Pedestrian | 2 |  |
|  |  | Forbidden | 1 |  |
|  | Touristic infrastructure | Present on a radius of 50 m | 4 |  |
|  |  | Absent on a radius of 50 m | 0 |  |
| Agricultural activities (A) | Permanent irrigation | <10% | 1 | 3 |
|  |  | 10% ‒ 25% | 2 |  |
|  |  | 25% ‒ 50% | 3 |  |
|  |  | 50% ‒ 75% | 4 |  |
|  |  | 75% ‒ 100% | 5 |  |
|  | Surface for agricultural use within the watershed | <20% | 1 | 5 |
|  |  | 20% ‒ 40% | 2 |  |
|  |  | >30% | 3 |  |
| The surface of the watershed (S) | N/A | <38,85 km^2^ | 1 | 1 |
|  |  | 38,85 km^2^ ‒ 155,39 km^2^ | 2 |  |
|  |  | 155,39 km^2^ ‒ 388,47 km^2^ | 3 |  |
|  |  | 388,47 km^2^ ‒ 1 942,35 km^2^ | 4 |  |
|  |  | >1 942,35 km^2^ | 5 |  |
| Ways of transportation (T) | Railways | Magistrale railways | 4 | 1 |
|  |  | Tourist railways or narrow gauge | 1 |  |
|  | Roads | Highways or bypass roads | 5 |  |
|  |  | National roads | 4 |  |
|  |  | County or local roads | 3 |  |
|  |  | Unpaved roads | 1 |  |
|  |  | No ways of transportation | 0 |  |
| Industrial activities (I) | Industrial activities | Present | 3 | 4 |
|  |  | Absent | 0 |  |
|  | Operational activities | Mines, quarries or dumps | 5 |  |
|  |  | Operating perimeters | 1 |  |
|  |  | No operating activity | 0 |  |
| Vegetation cover (C) | N/A | <5% | 5 | 1 |
|  |  | 5% ‒ 20% | 4 |  |
|  |  | 20% ‒ 35% | 3 |  |
|  |  | 35% ‒ 50% | 2 |  |
|  |  | >50% | 1 |  |

Source: Authors’ own representation.

**Table 4.** Criteria(indicators) used for calculation of HI index

| Parameter | Interval | Score |
| --- | --- | --- |
| Slope | First quartile | 1 |
|  | Second quartile | 3 |
|  | Third quartile | 4 |
|  | Fourth quartile | 5 |
| Slope exposition | The exposition favors the accumulation of pollutants | 5 |
|  | The exposition does not significantly affect the accumulation of pollutants | 3 |
|  | The exposition does not favor the accumulation of pollutants | 1 |
| Soil permeability | Clay soil (fine texture, low permeability) | 5 |
|  | Sandy soil (sandy texture, medium permeability) | 3 |
|  | Stones (coarse texture, high permeability) | 1 |

Source: Authors’ own representation.

**Table 5**. Data sources used for lakes delimitation and for the calculation of WRASTIC-HI index

| Source | Data type | Description | Use |
| --- | --- | --- | --- |
| Copernicus Land Monitoring Service | Corine Land Cover v.2012  (Poligon) | Double coverage of satellite images was used.  Mapping was done by computer assisted photo ‒ interpretation technology. Coverage: countries EEA39. | WRASTIC Index |
| Copernicus Paneuropean High Resolution Data | Permanent Water Bodies v.2012  (Poligon) | The data provides high-resolution categories of use. The delimitation of water bodies has been realized as a binary product (presence or absence) and includes permanent water bodies delimited with a spatial resolution of 20m. | Lakes delimitation |
| European Environmental Agency | EU‒DEM  (DEM) | EU ‒ DEM is a hybrid product, 1 degree resolution, based on SRTM and ASTER GDEM, weighted combined. The original reference system is ETRS89. The tiles were aggregated in 5°x5°, sections, redesigned in the ETRS ‒ LAEA system. | HI Index |
| European Environmental Agency | Major sources of pollution (vector) | The major sources of pollution are taken from the *European Pollutant Release and Transfer Register* (E ‒ PRTR), which contains reports of over 30000 high pollutant facilities covering 65 economic activities in EU countries, alongside Iceland, Liechtenstein, Norway, Serbia and Switzerland | WRASTIC Index |
| Open Street Map | OSM dataset  (Poligon) | Data are obtained through systematic field analyzes and entered into the OSM database. The availability of satellite data and photogrammetric images has led to a significant increase in the level of automation. | WRASTIC Index |
| National Agency for Mineral Resources | Perimetrele de exploatare  (Raster) | The map was made under the aegis of ANRM, following the conclusion of the agreements for exploration, development of natural resources located in any type of collector. | WRASTIC Index |
| Ministry of the Environment | SAC, SCI & SPA limits  (Poligon) | Delimitation of Special Conservation Areas (Habitats Directive) and Special Bird Protection Areas (Birds Directive), part of the Natura2000 network. | WRASTIC Index |
| European Soil Data Center | TSGD Eurasia  (Poligon) | The data were made for the use of the Land Resource Management (Institute for Environment & Sustainability) agencies within the EC Joint Research Center, developed in collaboration with the European Soil Bureau Network. | HI Index |
| European Environment Agency | Urban Waste Water Treatment,  Agglomeration – (Punct) | Information on the implementation of EU Directive 27‒ Urban Waste Water Treatment (UWWTD): the location of wastewater treatment plants, their compliance with the UWWTD directive and the type of water processing, the degree of processing in relation to the degree of wastewater generation. | WRASTIC Index |
| National Cadastre Agency and Real Estate Advertising | Administrative Boundry  st Order  (Poligon) | Data regarding the limits of UAT on the territory of Romania | Lakes delimitation |
| United States  Geological Survey | Landsat 8  (Raster) | Data with a resolution from 15 to 100 m for the entire land surface. Landsat 8 operates in visible, infrared, near infrared and thermal infrared spectrum. The program satellites record more scenes per day compared to Landsat 7. | Validation  of results and  control |

Source: Authors’ own representation.

**Table 6.** Soil permeability derived from the textural classes associated with the soil types

| **Category** | **Description** | **Score** |
| --- | --- | --- |
| Increased permeability | coarse textures, specific to the soils characterized by a predominance of the particles with dimensions over 0.02 mm (from coarse sand to lute sand) | 1 |
| Average permeability | medium textures, specific to the soils where the ratio between coarse and fine particles decreases from 5: 1 to 3: 1 (approximate ratios) those with medium dimensions keeping the ratio of about 2: 1 compared to coarse ones (from to sandy clay to dusty clay) | 3 |
| Low permeability | the fine textures, specific to the soils where the ratio between coarse and fine particles decreases to about 2: 1, and those with medium dimensions reach a concentration of 1: 1 compared to coarse ones (from clay to clay) | 5 |

**Table 7**. Reclassified values from the interval 0‒360.

| Plan | N | NE | E | SE | S | SV | V | NV | N |
| --- | --- | --- | --- | --- | --- | --- | --- | --- | --- |
| ‒1‒0 | 0‒20 | 20‒67,5 | 67,5‒112,5 | 112,5‒157,5 | 157,5‒202,5 | 202,5‒247,5 | 247,5‒ 292,5 | 247,5‒ 292,5 | 337,5  360 |

**Table 8**. The relationship permeability – slope.

| Slope | Permeability | Score |
| --- | --- | --- |
| 1 | 1 | 1 |
| 2 |  | 1 |
| 3 |  | 1 |
| 4 |  | 3 |
| 1 | 3 | 1 |
| 2 |  | 3 |
| 3 |  | 3 |
| 4 |  | 5 |
| 1 | 5 | 3 |
| 2 |  | 5 |
| 3 |  | 5 |
| 4 |  | 5 |

**Table 9.** Relationship permeability – slope – exposition.

| P ‒ P | Exposition | ∑ |
| --- | --- | --- |
| 1 | 1 | 1 |
|  | 3 | 1 |
|  | 5 | 3 |
| 3 | 1 | 1 |
|  | 3 | 3 |
|  | 5 | 5 |
| 5 | 1 | 3 |
|  | 3 | 5 |
|  | 5 | 5 |
